# Transcriptome analysis identifies pathways associated with enhanced maternal performance in QSi5 mice

**DOI:** 10.1186/1471-2164-9-197

**Published:** 2008-04-29

**Authors:** Palaniappan Ramanathan, Ian C Martin, Margaret Gardiner-Garden, Peter C Thomson, Rosanne M Taylor, Christopher J Ormandy, Christopher Moran, Peter Williamson

**Affiliations:** 1 Centre for Advanced Technologies in Animal Genetics and Reproduction, Faculty of Veterinary Science, University of Sydney, NSW 2006, Australia; 2 Cooperative Research Centre for Innovative Dairy Products, Faculty of Veterinary Science, University of Sydney, NSW 2006, Australia; 3 Garvan Institute of Medical Research, Darlinghurst, NSW 2010, Australia

## Abstract

**Background:**

Highly fecund mouse strains provide an ideal model to understand the factors affecting maternal performance. The QSi5 inbred strain of mice was selected for high fecundity and low inter-litter interval, and is very successful at weaning large numbers of offspring when compared to other inbred strains.

**Results:**

Post-natal pup weight gain was used to estimate mammary gland output and to compare the performance of QSi5 mice to CBA mice. Cumulative litter weights and individual pup weight gain was significantly higher throughout the first eight days of lactation in QSi5 mice compared to CBA mice. Morphometric analysis of mammary glands during pregnancy in QSi5 mice revealed a 150 percent greater ductal side branching compared to CBA mice (*P *< 0.001). Ontology and pathway classification of transcript profiles from the two strains identified an enrichment of genes involved in a number of pathways, including the MAPK, tight junction, insulin signalling and *Wnt *signalling. Eleven of these genes, including six genes from the MAPK signalling pathway, were identified as associated with postnatal growth. Further, positive mediators of Wnt signalling, including *Wnt4, Csnk2a1 *and *Smad4*, were over-represented in the QSi5 strain profile, while negative regulators, including *Dkkl1, Ppp2r1a *and *Nlk*, were under-represented. These findings are consistent with the role of Wnt and MAPK signalling pathway in ductal morphogenesis and lobuloalveolar development suggesting enhanced activity in QSi5 mice. A similar pattern of phenotype concordance was seen amongst 12 genes from the tight junction pathway, but a pattern did not emerge from the insulin signalling genes. Amongst a group of differentially expressed imprinted genes, two maternal imprinted genes that suppress growth induced *via *the IGF signalling pathway, *Grb10 *and *Igf2r*, were under-represented in QSi5 mice. Whereas *Peg3 *and *Plagl1*, both paternally imprinted genes that enhance neonatal growth, were over-represented in QSi5 mice.

**Conclusion:**

We propose that the combined action of at least three major signalling pathways involved in mammary gland development and milk secretion, namely Wnt, MAPK and tight junction pathways, contribute to the superior maternal performance phenotype in QSi5 mice. Additionally, favourable expression patterns of the imprinted genes *Peg3, Plagl1, Grb10 *and *Igf2r *may also contribute.

## Background

The capacity of highly fecund mouse strains to successfully reproduce is accompanied by a concomitant increase in maternal performance at sustainable litter sizes [1]. The increase in lactation demanded by larger litters can be met by a combination of physiological strategies that allow for enhanced mammary development, increased milk output and improved milk nutritional quality. The mechanisms that underpin these strategies are complex and are under the control of multiple regulatory pathways that may each contribute at various levels. An effective way to analyse these mechanisms is to identify key genes or regulatory pathways that influence lactation phenotype. Recently, functional genomic approaches have been successfully employed to identify genes that have altered expression during mammary gland development and initiation of secretion. As a result numerous gene candidates have been implicated during different stages of the lactation cycle with some mapped to distinct signalling pathways [2-4].

A complementary approach involves the comparison of mouse strains that have divergent phenotypes, in particular strains that are representative of the high and low extremes of the variation that exists in the mouse population. The QSi5 inbred strain of mice was established from an outbred Quackenbush-Swiss strain by full-sib inbreeding and selection on the basis of increased litter size and shortened inter-litter interval [5]. The strain has an average litter size greater than 13 pups, and females commonly nurse up to 18 pups with greater than 90% survival to weaning. Along with an increased body weight (BW), these traits are indicative of enhanced lactation capacity [6]. Indeed lactation performance, assessed by a weigh-suckle-weigh method, was 3-fold greater in QSi5 mice than in the CBA strain [1].

In this study, we utilised the divergent phenotypes of QSi5 and CBA/CaH (hereafter referred as CBA) mice to identify genes and associated pathways that correspond to enhanced mammary gland capacity. We identified a significant enrichment among the differentially expressed genes in the Wnt and MAPK signalling pathways, both of which are implicated as effectors of ductal side-branching during mammary gland development [2,7-9], and a finding that is consistent with anatomical differences found in the two strains. We also identified favourable expression patterns of genes in the tight junction pathway, and four imprinted genes that influence maternal performance in mice. We propose that the action of these signalling pathways and the effects of gene imprinting contribute to the superior maternal performance in the QSi5 strain.

## Results

### Maternal Performance

**Figure 1:**
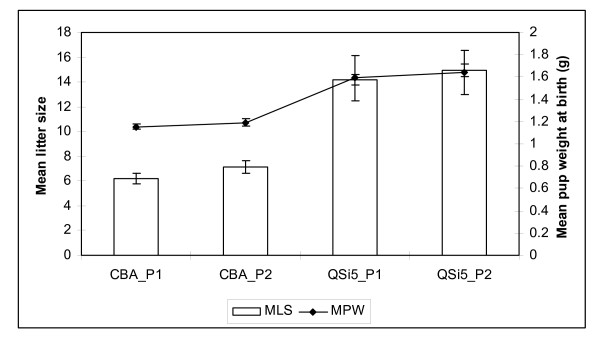
**Comparison of litter size and pup weight at birth in the two inbred strains**. oth number of pups born alive and mean pup weight at birth at the first two parities in CBA (n = 20) and QSi5 (n = 20) were significantly higher in the QSi5 strain of mice (p < 0.0001). Note: all error bars are + SEM.

We compared a highly fecund QSi5 inbred strain of mice to a non-selected CBA strain of mice raised on a similar genetic background for reproductive performance. The number of pups born alive (NBA) in the first and second parities respectively for QSi5 mice was 14.2 and 14.9 pups/litter, whereas for the CBA strain it was 6.2 and 7.2 pups/litter (Figure 1). Thus significant differences in fecundity exist between QSi5 and CBA strains of mice both in first and second parities (*P *< 0.001).

**Figure 2:**
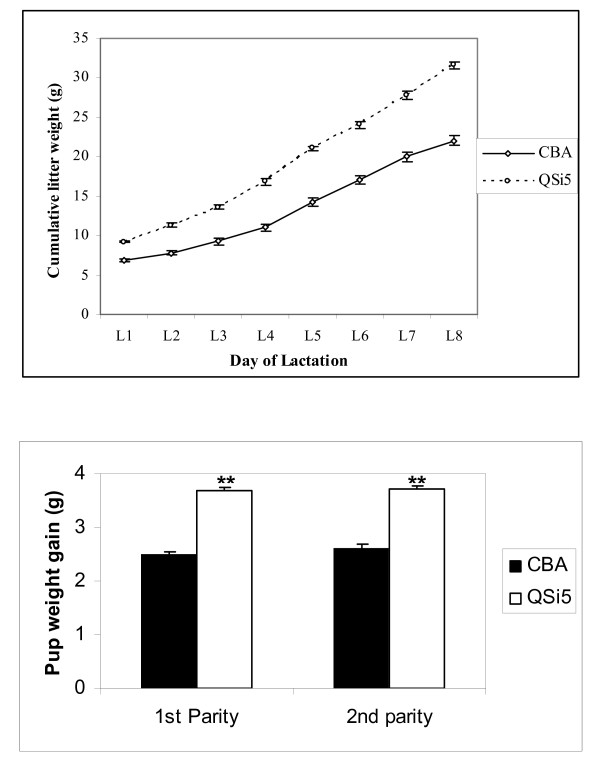
**Assessment of maternal performance in two inbred mice strains**. **(a) Cumulative litter weights in two strains **– Cumulative litter weights measured using 6 pups/dam during the first 8 days of lactation in CBA (n = 16) and QSi5 (n = 20) strains; Weights are reported as Mean ± SEM. Lactation performance was significantly greater in QSi5 dams compared to CBA. **(b) Individual pup weight gain at 8 days **– Mean pup weight gain at the first 2 parities in CBA (n = 16) and QSi5 (n = 20) strains of mice. Pup weight gain was significantly higher in QSi5 strain relative to CBA strain of mice in both parities.

To determine whether the increased maternal performance of QSi5 mice was independent of litter size we adjusted litter sizes on day of parturition for both the QSi5 and CBA strains, and calculated cumulative weights for a standardized six pup litter. On this basis, significant strain-specific differences in maternal performance (*P *< 0.001) were observed throughout the first eight days of lactation based on the cumulative litter weight (Figure 2a). Additionally, maternal performance assessed by individual pup growth rate was 150% greater (*P *< 0.001) in QSi5 mice when compared to CBA mice (Figure 2b). Further, there was no significant difference in pup growth rate between first and second parities within each strains. These results confirm that the superior maternal performance of the QSi5 mice was independent of the large litter size phenotype, and that significant differences in maternal performance exist between the QSi5 and CBA inbred strains of mice.

### Morphometric analysis

**Figure 3:**
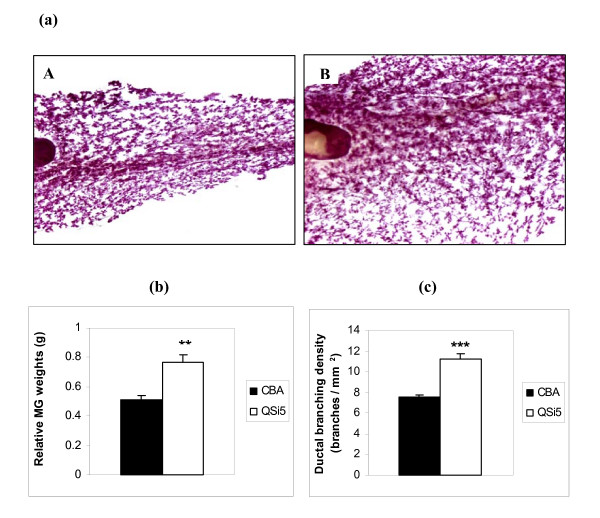
**Histological analysis of mammary glands from pregnant mice**. **(a) Whole mounts of the inguinal mammary gland from day 12 pregnant mice A. **CBA strain and **B. **QSi5 strain. **(b) Comparison of relative mammary gland weights of the two different strains **(n = 5 each). **(c) Quantitation of ductal branching **Both relative mammary gland weights (p < 0.001) and density of ductal branching (p < 0.001) were significantly higher in the QSi5 strain compared to CBA strain of mice.

To ascertain whether the strain-specific differences in maternal performance can be attributed to anatomical differences in the mammary gland during development, morphometric analysis of mammary whole mounts from mice at pregnancy day 12 (Figure 3a). When adjusted for body weight, a significant difference in relative mammary gland weight existed between the two strains (P < 0.001), calculated as 150% higher in the QSi5 strain relative to the CBA strain of mice (Figure 3b). Quantitation of ductal density in mammary gland whole mounts using image analysis software (NIH-ImageJ) revealed a significant difference in the proportion of ductal branching between the two strains of mice (P < 0.001) (Figure 3c). Ductal branching was approximately 150% higher in the QSi5 strain relative to the CBA strain. Similarly, relative quantitation of the terminal end buds (TEB) as an estimate of alveolar epithelial density showed a 140% increase in QSi5 mice compared to the CBA strain of mice (P < 0.0001). Thus, the QSi5 mice had both a greater mammary tissue mass and enhanced ductal side-branching; both consistent with a superior lactation phenotype.

### Mammary gene expression profiling

Gene expression profiling of mammary gland tissue is an effective approach to identifying pathways that are active during mammary gland development and lactation. To identify the genes associated with the enhanced maternal performance phenotype of the QSi5 strain, gene expression profiles of mammary gland tissue from five animals each of lactating QSi5 and CBA mice were compared. Lactation day 9 was chosen for quantitative measurement because neonatal growth is a better correlate of lactation performance during the plateau phase of milk production in mice, that is at 6–10 days post-partum [6]. Further, measurements on day 9 were comparable to other lactation cycle studies performed in mice [10].

**Figure 4:**
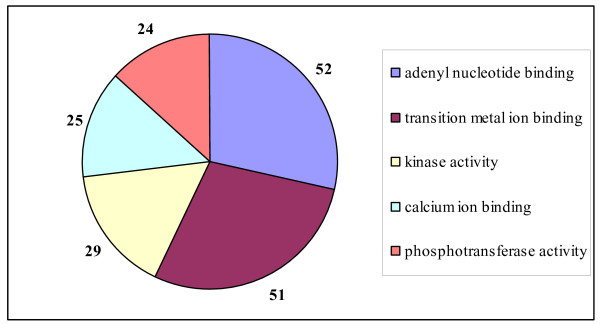
**Significant GO categories classified by DAVID**. Pie chart showing the significantly enriched molecular function categories (Level 5) based on gene ontological classification of the differentially expressed genes (p < 0.01) between the two strains of mice using DAVID.

We identified 740 probesets (640 genes) and 331 probesets (282 genes) using a permutation based FDR of 8.2%, and BH FDR of 5% respectively, as being differentially expressed between the two strains of mice. Gene ontology classification of the differentially expressed genes based on molecular function identified the dominant categories, including calcium ion-binding and kinase activity, to be significantly over-represented among the differentially expressed genes (Figure 4). The genes identified in the calcium ion-binding category, included some well-characterised genes associated with lactation, such as lactalbumin (*Lalba*), *Egf*, phospholipase C, epsilon 1 (*Plce1*), annexins in addition to Phospholipid scramblase 1 (*Plscr1*), phospholipase A2, group XIIA (*Pla2g12a*) and nucleobindins (*Nucb1, Nucb2*). Similarly, a number of known kinases associated with mammary gland development, including Nemo like kinase (*Nlk*), casein kinase (*Csnk1a1*), ribosomal protein S6 kinase, polypeptide 1 (*Rps6kb1*), and proviral integration site 1 & 2 kinases (*Pim1/2*). Importantly, positive mediators of mammary gland development such as *Lalba, Egf, Csnk1a1, Rps6kb1, Pim1 *all had higher levels of expression in the QSi5 mice relative to the CBA mice.

Gene ontology analysis of the differentially expressed genes based on the biological process category did not reveal any significant enrichment of carbohydrate and lipid metabolism. Subsequently, analysis of 37 genes corresponding to enzymes known to be involved in these metabolic processes [10] identified *Agpat1 *as the only gene to be differentially expressed.

**Figure 5:**
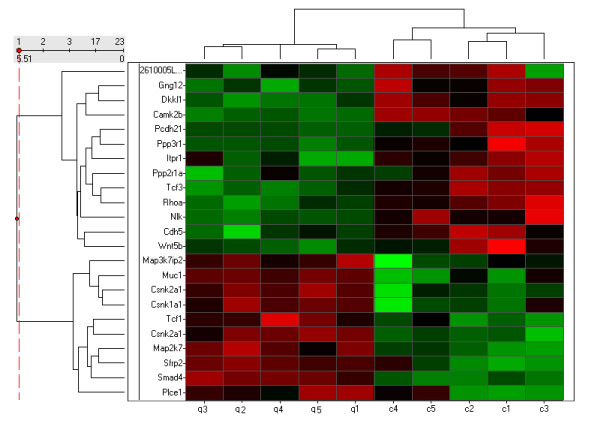
**Hierarchical clustering and heat map of differentially expressed Wnt signalling genes**. Clustering was performed on all ten transcript profiled samples (QSi5 – 1^st ^5 vertical columns and CBA – last 5 vertical columns) for ten genes up-regulated in QSi5 (bottom ten horizontal rows) and 15 down-regulated genes (top 15 horizontal rows) relative to the CBA strain of mice. Expression levels are colour coded with shades of red, green and black corresponding to an increase, decrease and no change in gene expression, respectively.

Pathway classification of the differentially expressed genes using the Onto-Express pathway analysis tool identified 16 genes in the MAPK signalling pathway, 12 genes in the tight junction signalling pathway and 10 genes each from the Wnt signalling pathway, cell adhesion molecules and insulin signalling pathways, as enriched among the differentially expressed genes (Table 1 and Additional file 1). Similar pathway analysis results were obtained when a group of 282 differentially expressed genes selected below a 5% FDR cut-off were analysed, and additionally included adherens, junction and TGF-beta signalling pathways. We also examined whether the differentially expressed genes in these pathways were associated with any of the mammary gland associated phenotypes found in the mouse genome informatics (MGI) database. Ten of the 48 genes in these pathways were associated with postnatal growth retardation or postnatal lethality, which is substantially influenced by maternal/lactation performance. This included seven genes, namely *Egf, Cdc42, Nlk, Jund1, Kras, Casp3 *and *Map3k12*, from the MAPK signalling pathway, a pathway that plays a critical role in mammary gland development *via *the EGFR response. Additionally, two genes that are associated with abnormal mammary gland development when deleted, *Egf *and *Neo1*, were both over-expressed in the QSi5 mice. Besides the ten unique Wnt signalling pathway genes identified in the above pathway analysis, five other genes closely associated with the canonical Wnt/β-catenin/TCF pathway, namely *Dkkl1, Muc1, Rps6kb1, Sdc 2 *and *Sdc 4*, were also differentially expressed between the two mouse strains. The Wnt pathway plays a significant role in initiation of mammary gland morphogenesis. Wnts are secreted glycoproteins which stabilize β-catenin by preventing its degradation by the PP2A-GSK3β-Axin complex, resulting in translocation of β-catenin to the nucleus where it activates downstream target genes. Further, all positive mediators of the canonical Wnt-β-catenin signalling pathway in this group, such as *Csnk1a1, Csnk2a1, Smad 4*, were over-represented, while negative regulators, including *Ppp2r1a, Cdh5 *and *Nlk*, were under-represented in the QSi5 mice relative to CBA mice (Figure 5).

**Table 1:** Enriched biological pathways among differentially expressed genes. Table showing the most enriched biological pathways (p < 0.1) based on pathway classification of differentially expressed genes (p < 0.01) between the two strains of mice using Onto-Express Pathway analysis tool. Rank, impact factor, p-values and the number of input genes associated with significant pathways are listed.

**Rank**	**Pathway Name**	**Impact Factor**	**# Input Genes in Pathway**	**p-value**
2	Tight junction	5.772	12	0.00
7	Insulin signaling pathway	3.197	10	0.04
9	MAPK signaling pathway	3.022	16	0.05
10	Cell adhesion molecules (CAMs)	3.002	10	0.05
14	Wnt signaling pathway	2.371	10	0.09

Amongst the 12 differentially expressed genes in the significantly enriched tight junction signalling pathway, seven and five genes were over- and under-represented respectively, in the QSi5 mice relative to CBA mice. Genes that play an important role in tight junction stability, including *Tjp2*, *Csnk2a1, Pard6g (Par6a), Llgl1 (Mgl1)*, were all over-represented in the high lactation performance QSi5 mice, whereas genes associated with tight junction permeability, namely *Ppp2r1a (PP2A) *and *Rhoa*, were over-represented in the low performance CBA mice. Thus the decreased tight junction permeability of the QSi5 mice may contribute to enhanced milk secretion. Also, amongst seven strain-specific differentially expressed genes in the TGF-β pathway,*Tgfb2 *and *Rhoa*, both negative regulators of mammary gland development, were over-represented in the low performance CBA mice relative to the QSi5 mice. However, despite over-representation in the gene ontology analysis, when genes belonging to the insulin pathway were examined there was insufficient evidence to conclude that a pattern of expression between the two strains reflected lactation performance.

Imprinted genes play a crucial role in facilitating transfer of nutrients from the mother to the offspring. Analysis of the differentially expressed genes identified four imprinted genes (Additional file 2). The paternally expressed genes, *Peg3 *and *Plagl1*, that are both growth promoting, were over-represented in the QSi5 strain relative to the CBA strain of mice. Conversely, two maternally expressed genes that are pup-growth limiting, *Grb10 *and *Igf2r*, were over-represented in the CBA mice.

The expression patterns of 17 milk protein genes previously identified as being differentially expressed in the mammary gland during the lactation cycle were also examined [10]. Five of these genes, namely Xdh, Muc1, Lalba, Egf, and Csn1s2a, were differentially expressed between the two strains and all were over-represented in the high lactation performance QSi5 mice. One other pathway known to be associated with mammary development and lactation, the prolactin receptor pathway was also examined. Key prolactin receptor-Stat5 signalling pathway encoding genes, such as *Sta5a, Stat5b, Prl, Prlr, Jak1, Jak2, or Socs1*, were not differentially expressed between the two strains. However, amongst the 25 Stat5 target genes identified in mammary epithelial cells [11], three genes, *Socs2, Pim1 *and *Grb10*, were differentially expressed. Of these *Grb10 *was over-represented in the CBA mice whereas the other two genes were over-represented in QSi5 mice.

Thus, differential expression of components of the tight junction, Wnt and MAPK signalling pathways together with favourable genomic imprinting may contribute to the increased maternal performance phenotype of the QSi5 strain of mice.

### Quantitative RT-PCR

**Figure 6:**
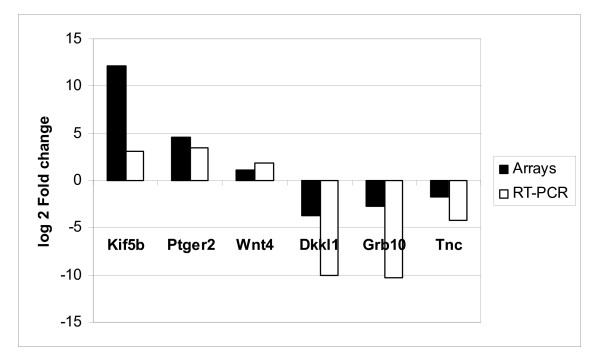
**Comparison of fold change differences in microarrays and qRT-PCR for selected genes**. Quantitative RT-PCR validation of the microarray results showing the observed fold change differences in both methods for three up regulated genes in QSi5 strain (*Kif5b*, *Ptger2*, *Wnt4*) and three down regulated genes (*Dkkl1*, *Grb10*, *Tnc*).

Quantitative RT-PCR (qRT-PCR) was used to evaluate the expression profiles of eight genes, including five genes identified as differentially expressed between the two strains on by microarray. In addition, *Wnt4 *was selected for validation as it was identified as differentially expressed based on the analysis of a subset of the data, and both *c-myc *and *CyclinD1 (Ccnd1) *were examined as potential target genes of Wnt signalling pathways. A significant degree of concordance, approximately 74% (p < 0.05) was observed between the two methods based on the expression patterns for all genes examined (Table 2). Six of the eight genes examined, namely *Kif5b, Ptger2, Grb10, Tnc *and *Dkkl1*, were differentially expressed between the two strains. Differential expression was not detected for the remaining two genes, *c-myc *and *Ccnd1*, but these genes are tightly regulated and are perhaps transiently expressed during stages of the cell cycle (Figure 6). Thus, the results of the qRT-PCR analysis generally supported those of the microarray data analysis.

**Table 2:** Quantitative RT-PCR validation of 8 selected genes.

**Gene Symbol**	**Microarrays**		**Real-time PCR**	
	
	**FC**	**P-value**	**FC**	**P-value**
Kif5b	12.17	< 0.001	3.14	0.005
Ptger2	4.58	< 0.001	3.42	0.01
Dkkl1	-3.76	< 0.001	-10.01	0.006
Grb10	-2.68	< 0.001	-10.32	0.09
Tnc	-1.76	0.001	-4.16	0.06
Wnt4	1.13	0.229	1.82	0.047
cyclin D1	1.08	0.66	1.95	0.12
cmyc	0.85	0.08	1.02	0.97

Comparison between array experiment and real-time PCR for 8 selected genes is shown. Log-fold change in real-time PCR was calculated from the difference in normalised expression between the CBA and QSi5 strains of mice. P-value was calculated from 2-tailed Student's t-test.

## Discussion

Highly fecund mice provide an opportunity to investigate the factors that control maternal performance. Comparison of the two phenotypically divergent inbred mouse strains, QSi5 and CBA, identified genes associated with maternal performance. Histological analysis of mammary whole mounts during mid-pregnancy revealed that QSi5 mice have increased ductal branching relative to the CBA strain of mice. Strain-specific differences in mammary gland architecture have been recognised for many years and may be regarded as a correlate of reproductive capacity. The differences may be due to epithelial factors [12,13], stromal factors [14], or hormonal factors [15]. Mammary gland recombination and transplantation experiments using mice from strains with highly branched architecture, the 129 and C57Bl/6J strains, which have a much lower level of side-branching, revealed that stromal factors were a likely source of variation [16], but differential expression of stromal genes has been difficult to demonstrate [17]. In the present study, a comparative analysis of gene expression in QSi5 and CBA mice identified candidate genes in a number of pathways, including tight junction, MAPK and Wnt signalling pathways, as contributors to the superior maternal performance phenotype of the QSi5 strain. Additionally, four imprinted genes that have a role in neonatal growth and mammary development, were identified as candidate genes that may also contribute to the phenotype.

Transcript profiling of mammary gland tissue from the two divergent strains during peak lactation revealed that Wnt signalling pathway genes were enriched within the differentially expressed genes. *Wnt *(wingless) genes are a family of secreted glycoproteins which commonly act through the canonical Wnt/β-catenin/TCF signal transduction pathway both in embryonic and postnatal mammary gland development [9,18]. Wnt genes, especially *Wnt4, Wnt5b *and *Wnt6*, are progesterone responsive and are expressed in a temporal pattern during pregnancy and lactation [19]. *Wnt4 *over-expression in virgin mice results in excessive ductal branching [20], while gene deletion reduces ductal side branching in mammary glands at pregnancy day 12 [21-23]. There is also evidence that different Wnt genes switch on or off at different stages of the lactation cycle [24]. The primary mode of action of the canonical *Wnt *signalling pathway is the stabilisation of β-catenin [25], which is necessary for normal lobular development of the mammary gland [8]. *Csnk1a1 *and *Csnk2a1*, which were over-represented in QSi5 mice gene expression profiles, are positive mediators of the pathway, while a lower level of expression was seen for *Ppp2r1a*, which has a central role in the β-catenin degradation complex. [26-28]. Thus, stabilization of cytoplasmic β-catenin may provide a mechanism to enhance lobuloalveolar development in the QSi5 strain of mice.

Postnatal growth of pups during the first two weeks is a key indicator of the lactation capacity of the dam. More than ten genes associated with this trait were identified amongst the enriched pathways. This included six genes of the MAPK pathway, namely *Egf, Cdc42, Nlk, Jund1, Kras *and *Map3k12*, which is activated during mammary gland development through the Egf receptor. Exogenous administration of Egfr ligands reinitiated ductal outgrowth during puberty in both ovariectomized mice [29] and *ER-α *knock out mice [30]. A role for the Egfr pathway in ductal development has been demonstrated by the phenotype observed in mice that have a functionally defective pathway [31,32]. There is also evidence that Wnts activate Egfr in murine mammary epithelial cells [33,34]. This is consistent with the findings of the present study, in which a relative enrichment of genes involved in both MAPK and Wnt signalling pathways was observed in QSi5 mice.

Decreased tight junction permeability results in enhanced milk secretion [35]. The significant enrichment of genes from the tight junction signalling pathway amongst those differentially expressed in the present study suggests that the pathway may play a role in the lactation performance differences between the two strains of mice. Positive mediators of the pathway include *Tjp2 *and *Csnk2a,1*, are involved inclaudin polymerisation and tight junction formation [36], and were over-represented in the QSi5 mice. Corresponding to this was a relative decreased expression of the negative mediators of this pathway, namely *Rhoa *and *Ppp2r1a*. Inhibition of *Rhoa *is required for tight junction formation in rat mammary epithelial cells [37]. Thus the potential for decreased tight junction permeability in QSi5 mice may contribute to enhanced milk secretion.

Imprinted genes play a critical role both in prenatal and postnatal growth, the latter being mainly influenced by the maternal performance of the dam. According to the "parent-offspring conflict hypothesis" paternally expressed imprinted genes favour the growth of its offspring and facilitates maximum transfer of nutrients from the mother, whereas maternally expressed imprinted genes inhibit growth and favour equal allocation of resources [38]. This hypothesis holds true even for the postnatal growth of offspring prior to weaning for most of the imprinted genes that have been identified in mammals [39]. In the present study, both the paternally-expressed imprinted genes, *Peg3 *and *Plagl1*, were over-represented in the superior performing QSi5 strain of mice whereas both the maternally-expressed imprinted genes, *Grb10 *and *Igf2r*, were under-represented. Previously it has been reported that pups raised on *Peg3 *deficient mice have reduced growth rate compared to their wild-type litter mates due to poor maternal care and impaired milk release [40]. *Peg3 *deficient offspring fostered on wild-type mothers had reduced birth weight and also had inferior growth rates compared to wild-type offspring [41]. *Plagl1 *deficient mice also produced offspring with reduced birth weight, and postnatal mortality was high, albeit difficult to interpret because of delayed lung maturity [42].

Both the maternally expressed imprinted genes *Grb10 *and *Igf2r *inhibit post-natal growth by blocking mitogenic effects of *Igf1 *and *Igf2 *[43-45]. *Grb10 *negatively regulates *Igf1 *[46] whereas *Igf2r *over-expression results in decreased cell growth and proliferation due to increased clearance of Igf2 levels as well as reduced phosphorylation of Igf1 receptor [47,48]. Over-expression of *Igf1 *results in prolonged lactation [49], while deletion of both *Igf1 *and *Igf1r *results in reduced ductal side branching and fewer TEB, respectively [50-52]. *Igf2 *has been shown to mediate prolactin induced alveolar development in the mammary gland and acts upstream of *cyclin D1 *during mid-pregnancy [53,54]. Hence, a relatively lower level of *Grb10 *and *Igf2r *gene expression is consistent enhanced maternal performance.

## Conclusion

To summarize, differential expression patterns of candidate genes in a number of pathways, including tight junction, MAPK and Wnt signalling pathways were associated with enhanced maternal performance of QSi5 mice relative to CBA mice. MAPK and Wnt pathways are both known regulators of ductal morphogenesis and alveolar development, while tight junction permeability is related to milk secretion,. Additionally, favourable expression patterns of imprinted genes in QSi5 mice may also contribute to a superior maternal performance phenotype.

## Methods

### Animals

Mice from QSi5 and CBA/CaH inbred strains were housed at the animal housing facility, Faculty of Veterinary Science, University of Sydney, and were maintained as per accepted guidelines. The room was maintained at a constant temperature of 21°C and the mice were exposed to a daily photoperiod of 12 hours (0600–1800). Mice had free access to water and a pellet diet (Rat and mouse cubes; Specialty Feeds, Glen Forrest WA 6071, Australia). 20 nulliparous QSi5 females and 19 nulliparous CBA females were mated to QSi5 and CBA males, respectively to assess lactation performance by neonatal pup growth method over the first two parities. Five nulliparous females of the QSi5 and CBA strains were similarly mated for transcript profiling of the mammary gland at day 9 of lactation, and five nulliparous females of each of the same strains were mated for histological analysis of mammary gland at day 12 of pregnancy.

### Assessment of maternal performance

Females of breeding age from each strain of mice were pair-mated at eight weeks of age and observed for successful mating for three consecutive days. The appearance of the vaginal plug was recorded as day 0 of pregnancy and the day of parturition as day one of lactation. At the first day of lactation, the number of pups born alive (NBA) were recorded and immediately litter sizes were adjusted. Both maternal and cumulative litter weights were recorded for the first eight days of lactation between 1000–1100 hours each day [55]. Statistical differences in cumulative litter weight gain between the two strains across lactation was estimated using a similar litter sizes of 6 pups/dam for both strains. Maternal performance in both first and second parities was assessed by estimating the individual pup weight gain between day 1 and day 8 of lactation. Statistical significance between the two strains was estimated by a Student's unpaired t-test.

### Histology and Morphometric Analysis

Left inguinal mammary glands harvested from day 12 pregnant mice were weighed and immediately used for preparing whole mounts. Mammary whole-mounts were prepared by spreading the mammary glands on a glass slide and fixing it in 10% formalin solution. Glands were treated in acetone before carmine alum (0.2% carmine, 0.5% aluminium sulfate) staining overnight. The whole-mount was dehydrated by using a graded ethanol series followed by xylene treatment for 60 min and storage in methyl salicylate [20]. Relative mammary gland weights for each animal were calculated by dividing the actual wet mammary gland weights by the maternal body weight. Quantitation of ductal side branching was performed by measuring the density of ductal branching using the NIH-ImageJ image analysis tool [56]. Similarly, TEB were counted as an estimate of alveolar density. Five representative fields of view from each gland were counted. Statistical differences between the two strains for relative mammary gland weights, ductal branching density and epithelial density were then estimated by a Student's unpaired t-test.

### Transcript Profiling

The inguinal mammary glands (right) harvested from a total of ten mice, five from each strain, at lactation day 9 and were subsequently used for mRNA isolation. Upon removal, the gland was immediately snap-frozen in liquid nitrogen before storing at -80°C until further use. Total RNA was extracted from 20 mg of mammary tissue using Qiagen RNeasy mini kit (Qiagen, VIC, Australia) according to the manufacturer's instructions. RNA quality, concentration and integrity of samples were ascertained using Agilent 2100 Bioanalyser (Agilent Technologies, Foster City, CA). The cRNA targets were generated as recommended by Affymetrix (Santa Clara, USA) and hybridized to MOE 430A 2.0 GeneChips (Affymetrix). The GeneChips were subsequently scanned in GeneChip Scanner 3000 and finally the probe intensities were obtained using GCOS operating software (Affymetrix). Quantile normalization and calculation of probe-set intensity was performed using the robust-multi array average (RMA) function available from the "affy" package in R [57]. Both the normalized data and the raw "cel" files from this experiment are available at the NCBI GEO website under the series record GSE9668. Data filtering removed low intensity probesets close to background noise by selecting probesets having an un-logged intensity of over 20 in at least one of the conditions. Differential expression was assessed by ranked penalised t-statistics on the logged data using lm.series and e-bayes function in the "limma" package in R [58]. *P*-values were adjusted for multiple testing by the Benjamini and Hochberg (BH) method using the "multest" package in R. Genes with an adjusted P-value less than 0.05 can be interpreted as having a false discovery rate of 5%. Alternately, when genes were classified as differentially expressed using a less stringent unadjusted p < 0.01 threshold, FDR was calculated by a permutation based method. Differentially expressed genes were classified according to the Gene Ontology functional category based on molecular function using DAVID (The Database for Annotation, Visualization and Integrated Discovery) [59].

### Pathway and mammalian phenotype ontology analysis

Pathway classification of the differentially expressed genes was performed using the Pathway Express tool available in Onto-Express [60]. Enriched pathways among the differentially expressed genes below a p-value of 0.1 were identified and those associated with mammary gland development are listed in Table 1. Hierarchical clustering of 25 differentially expressed Wnt signalling pathway genes and five replicate samples from each strain was performed using an Euclidean distance metric with average linkage (Spotfire DecisionSite 8.0) (Figure 5). Differentially expressed genes within these pathways were subsequently investigated for any mammalian phenotypes associated with maternal performance, such as post-natal growth retardation, post-natal lethality, mammary gland development and maternal/paternal imprinting by data mining the Mouse Genome informatics (MGI) database [61].

### Quantitative RT-PCR

First strand cDNA synthesis was performed using MMLV reverse transcriptase (Invitrogen, VIC, Australia) according to manufacturer's instructions. PCR primers for all eight target genes used for validation and one housekeeping gene *Cyclophilin A (CypA) *were designed based on 100% homology to the RefSeq as well as mismatch to other genes. PCR primer (Sigma Genosys, Castle Hill, NSW, Australia) sequences and the product sizes are listed in Table 3. Quantitative PCR was performed using a master mix containing Platinum Taq DNA Polymerase (Invitrogen, VIC, Australia), 2.5 pmol of each primer and Sybr Green I (Molecular Probes, Eugene, OR, USA) using a Rotor-Gene 6000 real-time rotary analyzer (Corbett Life Science, NSW, Australia). Each reaction was performed in duplicate using a minimum of four samples from each strain and using cDNA from at least two separate cDNA synthesis reactions. Relative quantitation of the PCR products was performed by normalizing to an internal control, *CypA*. Statistical significance was determined by using Student's unpaired *t*-test and the observed fold-change differences between the two strains are reported.

**Table 3:** Primer sequences used in real-time PCR. Table showing the forward (F) and reverse (R) primer sequences for 8 target genes and one housekeeping gene used in real-time PCR along with the amplicon sizes.

**Name**	**Primer sequence**	**Product size (bp)**
***Kif5b *F**	GCGGAGTGCAACATCAAAGTG	127
***Kif5b *R**	CATAAGGCTTGGACGCGATCA	
***Ptger2 *F**	ATCACCTTCGCCATATGCTC	152
***Ptger2 *R**	GGTGGCCTAAGTATGGCAAA	
***Grb10 *F**	GCCAAGCATGATGTCAAAGTCT	183
***Grb10 *R**	GTCCTCCAGGCACCTCTCTA	
***Dkkl1 *F**	ACTGAGGGTCTTGCTGCTGCT	218
***Dkkl1 *R**	GGAAGTTCCTAGGAAGGTCTC	
***Tnc *F**	ACGGCTACCACAGAAGCTG	247
***Tnc *R**	CGCGGCTTATTCCATAGAGTTC	
***Wnt4 *F**	CGAGGAGTGCCAATACCAGT	138
***Wnt4 *R**	GCCACACCTGCTGAAGAGAT	
***C-myc *F**	CACCAGCAGCGACTCTGAA	99
***C-myc *R**	CCCGACTCCGACCTCTTG	
***Ccnd1 *F**	CATCAAGTGTGACCCGGACTG	116
***Ccnd1 *R**	CCTCCTCCTCAGTGGCCTTG	
***CypA *F**	GAGCTGTTTGCAGACAAAGTTC	125
***CypA *R**	CCCTGGCACATGAATCCTGG	

## Authors' contributions

PR, IM and PW designed and implemented the study. PW and RT supervised the study and prepared the manuscript with PR. MGG and PT contributed expertise and guided PR's analysis of microarray data. CJO and CM provided resources to enable the study. All authors had direct input to the final version of the manuscript.

## Supplementary Material

Additional file 1 **Gene lists of enriched biological pathway categories**. Strain-specific differentially expressed genes between the QSi5 and CBA strains of mice belonging to the five enriched biological pathway categories are listed along with their P-values and fold changes. Click here for file

Additional file 2 **Differentially expressed imprinted genes**. Strain-specific differentially expressed imprinted genes between the QSi5 and CBA strains of mice are listed along with their P-values and fold changes. Click here for file
